# In vitro comparison of microleakage with two different techniques of placing stainless steel crowns on mandibular deciduous first molar teeth with decreased mesiodistal width

**DOI:** 10.34172/joddd.2022.006

**Published:** 2022-05-29

**Authors:** Mahsa Sayadizadeh, Raziyeh Shojaeipour, Hamidreza Poureslami, Sajad Raeisi Estabragh, Maryam Sharifi

**Affiliations:** ^1^Department of Pediatric Dentistry and Oral and Dental Diseases Research Center, Kerman University of Medical Sciences, Kerman, Iran; ^2^Department of Pediatric Dentistry, Dental School, Kerman University of Medical Sciences, Kerman, Iran; ^3^Department of Prosthodontics and Oral and Dental Diseases Research Center, Kerman University of Medical Sciences, Kerman, Iran

**Keywords:** Crown, Deciduous teeth, Leakage, Stainless steel drown

## Abstract

**Background.** Stainless steel crowns (SSCs) of the opposing maxillary deciduous molar teeth are used in mandibular deciduous first molars with decreased proximal surfaces due to caries. However, the SSCs of maxillary deciduous molar teeth are different from those of the mandibular deciduous molars in terms of the occlusal surface morphology, the buccal margin, and the proximal surface contour. Therefore, it is possible to prepare the buccal and lingual surfaces to use the SSC of the lower deciduous molar teeth and compare microleakage.

**Methods.** Eighty extracted mandibular deciduous first molars were randomly assigned to two groups. In the case group (BLP), the buccal (B) and lingual (L) surfaces were prepared in addition to the proximal (P) surface, and an SSC was placed on the mandibular first deciduous teeth. Only the proximal surface was prepared in the control (P) group, and the SSC of the opposing tooth (maxillary deciduous first molar teeth) was placed. After dissecting the teeth, the extent of dye penetration was measured.

**Results.** The difference in microleakage on the buccal aspect between the case and control groups was significant (*P*=0.02); however, the difference in microleakage on the lingual aspect between the case and control groups was not significant (*P*=0.89).

**Conclusion.** Microleakage at the buccal margin of the SSC of mandibular deciduous first molars was less than the maxillary deciduous first molar SSC, with no significant differences in the lingual margin.

## Introduction

 Stainless steel crowns (SSCs) were introduced by Humphrey in 1950 for restoring deciduous molar teeth^1-6^ and are one of the most common treatment options during the deciduous dentition period.^7^ It is very difficult to achieve a proper marginal seal in SSC due to limitations in adjusting the size and shape of these prefabricated crowns,^8^ leading to microleakage at the margins due to inadequate marginal adaptation.^1^

 The marginal seal of SSCs depends on selecting a proper size, trimming the margins to achieve an appropriate length, crimping the margins to approximate them to the prepared tooth surfaces, and adequate polishing.^9^ The techniques used to prepare the tooth, too, affect microleakage.^10,11^ In proximal caries, the adjacent tooth usually moves to the space usually occupied by a sound tooth; therefore, the SSC is too big mesiodistally to be adapted to the buccolingual dimension of the tooth.^12^ If the lost space is on the distal aspect of the mandibular deciduous first molar tooth, usually the SSC of the opposing (maxillary) deciduous first molar tooth is used for the crown’s proper size.^13^

 SSCs are the most commonly used restorations in the mandibular deciduous first molars due to their unique morphology, including the convergence of the buccal and lingual surfaces toward the occlusal surface, the narrowness of the food table, and the large area of the contact surface with the deciduous second molar tooth. In addition, in most cases, the SSCs of the opposing tooth are used since the mesiodistal dimension of the tooth is lost due to caries. It should be emphasized that the occlusal surface morphology of the SSC of maxillary and mandibular first deciduous teeth and their gingival margins on the buccal aspect are different. Therefore, microleakage, which is the most important reason for restoration failure,^14^ should be compared when the mandibular deciduous first molar is prepared to receive the SSC of the opposing tooth with that when this tooth is prepared to receive the SSC of the mandibular deciduous first molar tooth. It is also necessary to determine which SSC is better for a mandibular deciduous first molar that has lost its mesiodistal width due to caries.

## Methods

 The present in vitro study was carried out on 80 extracted mandibular deciduous first molars in the Oral and Dental Research Center, Kerman University of Medical Sciences, Kerman, Iran, from September 2017 to December 2018.

 Mandibular deciduous first molar teeth extracted in several treatment centers due to systemic conditions, excessive loss of the bone support, the involvement of the permanent tooth, or the child’s lack of cooperation to render treatment were stored in normal saline solution. After obtaining informed consent from the parents, 80 teeth were selected from 112 extracted teeth according to the following inclusion criteria.

1. Intact mesial, buccal, or lingual surfaces or with minimum caries; decreased mesiodistal dimension due to caries on the distal aspect
2. Adequate root length for mounting the tooth in acrylic resin

The teeth were cleaned with a rubber cup and pumice powder to remove soft debris from the tooth surfaces. The pulp tissue was eliminated, and the access cavity was sealed with amalgam. The teeth were randomly assigned to two groups (n = 40):

*The case (BLP) group:* In addition to the proximal surface (P), the buccal (B) and lingual (L) surfaces, too, were prepared, and mandibular SSC was placed.

***The control (P) group:*** Only the proximal surface was prepared, and a maxillary SSC was placed.

 In both groups, the occlusal surface was reduced 1–1.5 mm with a wheel bur (Teezkaran Ltd; Tehran, Iran), and the proximal undercuts were eliminated with a feather edge bur (Teezkaran Ltd; Tehran, Iran). The occlusal third of the buccal surface was beveled with a wheel bur. In the BLP group, after eliminating the proximal undercuts, the buccal and lingual surfaces were prepared to achieve a rectangular cross-section of the tooth that resembled the mandibular deciduous first molar tooth. The angels were rounded in both groups. The SSC size (3M ESPE, St. Paul, MN) in both groups was selected with trial and error. After adaptation, if the retention was not satisfactory, similar to the clinical procedures, contouring and crimping were carried out (no. 114, 3M ESPE, and no. 800−417, Denovo, Baldwin Park, Calif), and the tip of an explorer was used to verify a proper adaptation. Then, two-thirds of the SSC was filled with cement (GC American, Inc., Alsip, III). The crown was seated with finger pressure and held in place with 5-kg axial pressure for the cement’s complete setting with a mastication simulator. The teeth were then mounted in acrylic resin with the crown completely exposed. One hole (as a reference) was created in the middle of the lingual surface of the teeth mounted in the acrylic resin (this point was the middle of the lingual surface where the SSC margin ended).

 A postgraduate student in pedodontics carried out all the tooth preparation steps, selected the SCCs with the proper size, adjusted them, and cemented them in both groups. The cement was mixed according to the manufacturer’s instructions. Excess cement was removed with an explorer, and the samples were stored at 100% relative humidity at 37ºC for 50 minutes and then incubated in deionized water at 37ºC for four weeks. The teeth underwent a 1000-round thermocycling procedure at 5–55ºC in a water bath with a dwell time of 30 seconds and a transfer time of 20 seconds. The samples were immersed in a 2% basic fuchsine solution for 24 hours and then dissected from the reference point in a BL direction with a diamond desk (Dorsa, HLF86, Tehran, Iran). Some of the teeth were lost during dissection due to the separation of the SSC or fracture of a part of it, leaving 37 teeth in the BLP group and 30 teeth in the P group.

**Figure 1:**
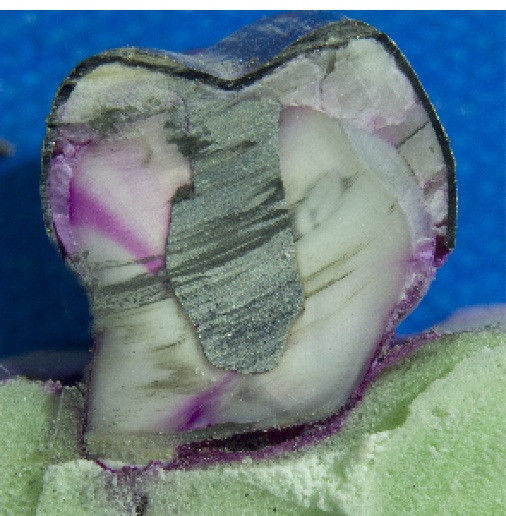
Microleakage values in the case group.

**Figure 2:**
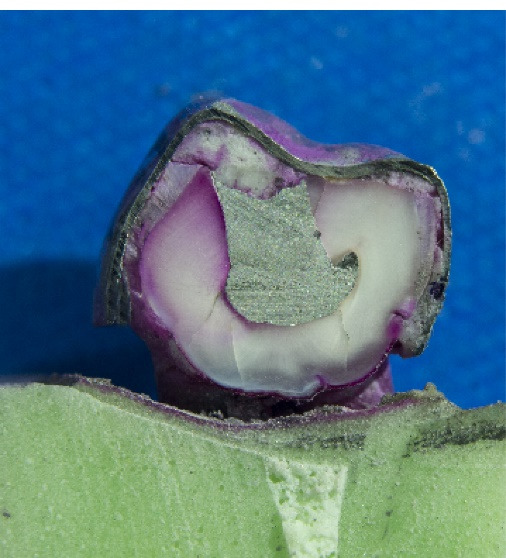
Microleakage values in the control group.

 The dye penetration (microleakage) from the SSC margin along the buccal and lingual surfaces in the mesial half of the dissected teeth was blindly measured in mm with a Vernier caliper by three independent viewers under a stereomicroscope (Technica, Germany) at ×40 magnification. An assistant handed in the teeth to the viewers for blinding. Figures 1 and 2 present the microleakage values in the case and control groups, respectively.

 The data were analyzed with SPSS 20. The means and standard deviations for all the three numeric values measured by the three reviewers were calculated on the buccal and lingual surfaces of each tooth.

 Mann-Whitney U test was used to compare microleakage on the buccal and lingual surfaces in the two groups. Since the data were distributed normally in the control group, a paired-samples *t* test was used to compare microleakage between the buccal and lingual surfaces. Since the data were not distributed normally in the case group, the Wilcoxon test was used for statistical analysis. Friedman’s test was used to compare the values recorded by the three viewers. A 95% confidence interval was considered.

## Results

 There were no significant differences between the mean microleakage values recorded by the three reviewers on the buccal surface in both groups (case group: *P* = 0.16, control group: *P* = 0.55). In addition, there were no significant differences on the lingual surface in the case group (*P* = 0.55); however, in the control group, there were significant differences between the means recorded by the three reviewers (*P* = 0.01).

 The mean ± SD of microleakage in the case group on the buccal surface was 3.11 ± 1.77 mm, with 3.48 ± 0.8 mm on the lingual surface; the mean ± SD of the mean microleakage in the control group on the buccal surface was 3.52 ± 0.83, with 3.45 ± 0.74 mm on the lingual surface.

 The difference in microleakage on the buccal surface was significant between the case and control groups (*P* = 0.02); however, the difference in microleakage on the lingual surface between the case and control groups (*P* = 0.89), the difference in microleakage between the buccal and lingual surfaces in the case group (*P* = 0.003), and the difference in microleakage between the buccal and lingual surfaces in the control group (*P* = 0.72) were not significant.

 The data were classified into three categories, as follows to show the severity of microleakage:

- Grade I: Microleakage up to 1–2 mm
- Grade II: Microleakage up to 2–3 mm
- Grade III: Microleakage up to 3–5 mm

According to Table 1, the frequencies of the three microleakage grades on the buccal aspect of the case group were as follows: grade I: 4 teeth; grade II: 20 teeth, and grade 3: 13 teeth; on the lingual surface: grade I: no teeth; grade II: 12 teeth, and grade III: 25 teeth.

**Table 1:** The frequency distributions and percentages of microleakage grading on the buccal and lingual surfaces in the case and control groups

**Microleakage grading**	**Case group**		**Control group**	
	**Buccal surface**	**Lingual surface**	**Buccal surface**	**Lingual surface**
Grade I	4 (10.8)	0 (0)	1 (3.3)	2 (6.2)
Grade II	20 (54.1)	12 (32.4)	9 (30)	9 (30)
Grade III	13 (35.1)	25 (67.6)	20 (66.7)	19 (63.3)

n(%): the number of the teeth in each grade (the percentage of the number of the teeth with grade I, II or III in each subgroups)

 The frequencies of the grades on the buccal aspect in the control group were as follows: grade I: 1 tooth; grade II: 9 teeth; and grade 3: 20 teeth; on the lingual surface: grade I: 2 teeth; grade II: 9 teeth; and grade III: 19 teeth.

 Considering the *P* values of Fisher’s exact test presented in the above table, it might be concluded that there was a significant relationship only between the buccal surface microleakage in terms of the crown type (the control group vs. the case group) (*P* = 0.03). However, there was no significant relationship between the lingual surface and the crown type (*P* = 0.37).

## Discussion

 SSCs are used for the definitive restoration of deciduous molar teeth.^15-17^ They have a significant role in the development of occlusion and mastication by reconstructing the crowns of deciduous teeth and are the most durable restorations to preserve the deciduous teeth until they exfoliate. On the other hand, the most important reason for the failure of restorative materials is microleakage due to dimensional and thermal changes, mechanical stresses, or a lack of adaptation of the restorative material with the tooth structure, leading to the early loss of the restoration or endangering its longevity; it also provides access for the bacteria to the pulp chamber, resulting in treatment failure.^3^

 The present study evaluated one of the factors affecting microleakage in SSC restorations, which is used to prepare the teeth. The gaps between the SSC and the prepared tooth structure were evaluated in two groups with different preparation methods of mandibular deciduous first molars and compared.

 The morphologies of the occlusal surfaces of mandibular and maxillary deciduous first molars are different. Therefore, when the mandibular deciduous first molars are a candidate for using the SCC of the maxillary deciduous first molar, the lower tooth should undergo more preparation on the buccal and lingual surfaces to receive the SSC of the opposing tooth, which increases microleakage. In the case (BLP) group, preparation was carried out on the buccal and lingual surfaces, and the mandibular SSC was placed. In the control group (P), no preparation was carried out on these two surfaces, and the maxillary SSC was placed. The results showed significantly lower microleakage on the buccal surface in the case group than the control group, with no significant difference on the lingual surface.

 The significant difference on the buccal surface might be attributed to the unique anatomy of the mandibular deciduous first molar and the SSC’s shape. The cervical ridge on the deciduous first molar’s buccal surface was removed during preparation in the case group; however, such preparation was not carried out in the control group. Therefore, the distance between the SSC and the tooth was minimal in the case group, resulting in a significant difference in microleakage on the buccal surface.

 In the present study, microleakage was observed in all the samples, which was not unexpected considering the prefabricated nature of SSCs, resulting in a lack of complete adaptation in the margin area, despite contouring and crimping. Moreover, in addition to measuring the extent of microleakage, the two groups were graded in terms of the microleakage severity and compared. Grade II microleakage exhibited the highest frequency on the buccal surface in the case group, with grade III as the most frequent grade in the control group. On the lingual surface in both groups, grade III was the most frequent one.

 In contrast to the present study, in a study by Seraj et al,^3^ the remaining tooth structure did not affect microleakage severity in restorations with SSCs. In the in vitro study above, 30 extracted deciduous molar teeth were assigned to two groups of intact and carious. After preparing the teeth, SSCs were cemented. After the laboratory procedures similar to the present study, it was concluded that microleakage was not affected by the severity of crown destruction. The discrepancy between the present study results and those of the study by Seraj et al can be attributed to the methodology; in the present study, the teeth in both groups were similarly carious. In addition, the caries was mild and only on the distal surface, and the three other surfaces (buccal, lingual, and mesial) were sound. However, the similarity between the present study and that by Seraj et al was microleakage in all the samples, indicating a lack of complete adaptation of the marginal area in prefabricated SSCs.

 In a study by Yilmaz et al,^18^ SSCs were placed on 63 deciduous first molars, and microleakage was evaluated under a microscope. They reported that an increase in SSC retention was associated with less microleakage. In a study by Subramaniam et al,^19^ SSCs were placed on 45 deciduous first molars, and it was concluded that the retention of SSCs on the remaining tooth structure affected the treatment success. The two studies above, similar to the present study, showed that the tooth preparation method affected SSCs’ marginal seal. Veerabadhran et al^20^ created a transverse retentive groove in the middle third of the buccal surface of 32 deciduous second molar teeth and restored the teeth with SSCs, finally reporting that the tooth preparation method did not affect SSC’s retention.

 Myers et al^21^ reported that the cervical area of a deciduous tooth has a significant role in the retention of SSCs, and if the cervical area of the tooth is not prepared, the retention of the SSC is not affected by the remaining tooth structure. In the present study, too, in the case group in which the buccal and lingual surfaces were prepared, compared to the control group in which these two surfaces were not prepared, SSC retention was better, and microleakage was lower, consistent with the study by Myers.

 Sohrabi et al^14^ evaluated microleakage in terms of the tooth preparation method and concluded that teeth with minimal preparation, restored with a jacket crown, exhibited more microleakage than teeth restored with conventional SSCs. The present study, too, showed that teeth with more preparation receiving the mandibular deciduous first molar crown exhibited less microleakage on the buccal surface than teeth with less preparation with the crown of the opposing arch.

 In vitro, microleakage studies are an essential initial screening method for assessing restorative materials’ sealing ability in the clinic. The dye penetration technique has been used in many studies evaluating marginal seal.^10,22^ In the present study, too, microleakage was assessed with the penetration of fuchsine dye, which is the most widely accepted technique for evaluating microleakage. The advantages of using coloring solutions include accurate evaluation of the marginal seal, the direct observation of the diffused marker under a microscope, and their easy use. This technique’s disadvantage is the small particle size of the coloring agent compared to the size of bacteria, resulting in dye penetration in all the samples.^10^ In studies evaluating microleakage, dye penetration is usually reported in percentage. In the present study, dye penetration was measured in millimeters to minimize errors in reporting percentages or proportions using approximate values.

 The SSC of the mandibular deciduous first molar tooth is different from that of the maxillary deciduous molar tooth in terms of the occlusal surface anatomy and proximal surface contours. Therefore, clinically, the odds of premolar contact with the opposing tooth, the presence of a different embrasure in contact with the deciduous second molar tooth, and closure of the primate space are not unexpected after placing the SSC of the opposing tooth on the deciduous first molar tooth, leading to the idea of placing the SSC on the deciduous first molar tooth after preparing the buccal and lingual surfaces in teeth with reduced proximal surfaces. The present in vitro study showed that placing the SSC of the mandibular deciduous first molar instead of that of the opposing tooth might decrease microleakage. Therefore, further in vitro studies are suggested to evaluate this issue and other important issues, such as the retention of SSCs. This method should also be evaluated clinically to assess the three factors of premature contact, embrasure status, and preservation of the primate space.

## Conclusion

 The present in vitro study showed that when a mandibular deciduous first molar tooth with decreased proximal surfaces is a candidate to receive the opposing jaw’s SSC, it is possible to prepare the buccal and lingual surfaces of the teeth to use its own SSC. In such a case, microleakage at the SSC’s buccal margin is less than that of the upper SSC, with no significant difference at the lingual margin.

## Acknowledgments

 The authors thank Mr. Ali Karamoozian, a Ph.D. student of Biostatistics, Department of Biostatistics and Epidemiology. The authors also thank the parents who signed informed consent forms so that their children could be included in the study. The authors also thank the Oral and Dental Research Center of Kerman University of Medical Sciences and their staff.

## Authors’ Contributions

 MS: experimental studies, data analysis, the definition of intellectual content; RS: concept, design, manuscript review, manuscript preparation, statistical analysis; HP: concept, design, manuscript review, manuscript editing; SR: experimental studies, literature search, manuscript preparation; MS: data analysis, literature search.

## Funding

 The study was financially supported by the Oral and Dental Research Center of Kerman University of Medical Sciences.

## Ethics Approval

 The study protocol was approved by the Ethics Committee of Kerman University of Medical Sciences under the code of IR.KMU.REC.1396.1398.

## Competing Interests

 The authors have declared that no conflict of interest exists.
